# Pupils' participation roles in school-based physical activity in the context of physically active learning and recess: experiences from Norwegian and Estonian primary and secondary school pupils

**DOI:** 10.3389/fspor.2025.1514764

**Published:** 2025-02-24

**Authors:** Eirini Pardali, Lise Kjønniksen, Solfrid Bratland-Sanda, Egle Säre, Ingrid Koni, Getter Marie Lemberg, Evelin Mäestu, Merike Kull, Mathias Brekke Mandelid, Lise Katrine Jepsen Trangsrud

**Affiliations:** ^1^ Department of Sports, Physical Education and Outdoor Studies, University of South-Eastern Norway, Bø i Telemark, Norway; ^2^ Department of Sports, Physical Education and Outdoor Studies, University of South-Eastern Norway, Notodden, Norway; ^3^ Institute of Education, University of Tartu, Tartu, Estonia; ^4^ Institute of Sport Sciences and Physiotherapy, University of Tartu, Tartu, Estonia; ^5^ Center for Physically Active Learning, Department for Pedagogy, Religion and Social Studies, Western Norway University of Applied Sciences, Bergen, Norway

**Keywords:** school-based physical activity, participation roles, physically active learning, recess, Hart's ladder of participation

## Abstract

**Introduction:**

The role of pupil participation in school-based physical activity is an often overlooked element despite the increasing interest in pupils' voices in policy development and research. Pupil participation is here defined as collaborative actions involving pupils and school stakeholders that influence decision-making processes in school-based physical activity. This study was conducted to explore how the 10- and 15-year-old pupils perceive their participation roles in school-based physical activity connected to age and the contexts of physically active learning and recess in various schools in Norway and Estonia.

**Methods:**

The study involved thirty-eight students (*n* = 38; 17 boys and 21 girls; 21 10-year-olds and 17 15-year-olds) from selected schools in Norway and Estonia. Data were gathered through nine semi-structured focus group discussions.

**Results:**

Utilizing Braun and Clarke's reflexive thematic analysis, three principal themes were constructed: 1. *The informed and listened to*, 2. *The responsible and open to teachers' questions and facilitation*, and 3. *The invited to make a choice and practice participation*. Taken together, the themes reflected the existing practices and structures that empowered pupils to express their perspectives on school-based physical activity. They also encapsulated pupils' suggestions and wishes for facilitation of their voices in these activities.

**Discussion:**

Using Hart's Ladder of Children's Participation as a theoretical lens, the study revealed age and context-specific disparities in pupils' participation roles in school-based physical activity. Pupils engaged in both informal and formal participation structures in teacher-led physically active learning and free choice recess activities, creating democratic spaces for rights of participation and the expression of choice, which need to be prioritized. Older pupils had more participation opportunities than 10-year-olds and had more responsibility, possibly due to perceived maturity. However, 10-year-olds exhibited numerous ideas for increased movement in school, valued the pupils' council, and called for more physical activity. Activity choice and teacher facilitation are vital in physically active learning and recess contexts. It is suggested that school-based physical activity can offer a space for pupils to learn about democracy but gaining the confidence and ability to do so takes practice. Meanwhile, teachers often need training to support active pupil participation in school-based physical activity.

## 1 Introduction

Following the adoption of the United Nations Convention on the Rights of the Child (UNCRC) in 1989 (1), children have the right to express their views and be listened to in matters that affect them. Ever since the introduction of the Ottawa Charter (2) and subsequent amendments, participation has been recognized as a crucial aspect in the accomplishment of health promotion goals. These goals include the empowerment of individuals and communities, as well as addressing social determinants of health and individual lifestyles (3). Meaningful participation of children lies at the heart of health promotion, with schools appearing to be the ideal setting for the promotion of both physical activity (4) and participation (5). Pupils have unique perspectives on health habits and health behaviour, which are valuable for tailoring interventions to their needs, thus potentially increasing the effectiveness and sustainability of programs (6). In school-based physical activity, participation can be defined as actions that influence decision-making about designing, planning, implementing and/or evaluating physical activity measures based on collaboration between pupils and school actors (7). The focus of this study is the pupils' perceived participation roles in school-based physical activity in two different contexts during the school day: (1) physically active learning (PAL) during regular classes and (2) recess. Here, PAL is examined as (i) active lessons, where “lesson content and learning occurs through engaging in movement and physical activity” (8, p. 1) and (ii) active breaks, involving “physical activity used as a stand-alone activity that provides a break from academic instruction, within or between lessons, with no curricular content included” (8, p. 1). The second context under examination in this study is recess time. Recess is considered as autonomous and free play time for pupils away from lessons, providing increased levels of physical activity and fitness, enhanced classroom attention, better cognitive abilities and learning skills and improved social and emotional skills (9). In this study, participation is not only taking part in activities or solving tasks, such as in PAL, but it also includes pupils' influence on the decisions and activities in collaboration with other stakeholders (3).

The involvement of pupils' voices in school-based physical activity can have numerous individual, organizational and societal benefits. On an individual level, such involvement can help improve pupils' life skills and motivation and make them more engaged in issues of public concern (10). Other benefits include legal responsibility, improvement of services, strengthening of decision-making and empowerment (11). At the organizational level, pupil involvement can lead to changes in school culture, rules, curriculum, and infrastructure, and improved school engagement (3). However, pupil participation can entail some undesirable outcomes. For instance, pupils from families of higher socioeconomic status are more likely to take part and direct the participatory processes, thus widening social inequalities (12, 13). Another negative result is that although studies of participation report youth advocacy as one of the outcomes, they rarely follow up to ascertain whether the proposed changes actually occur and are sustainable (14).

The UNCRC maintains that children have the right to articulate their opinions and be heard. Although both the Norwegian and Estonian curriculum are governed by the UNCRC (1), the practical application of this principle can differ. The 2020 Norwegian curriculum emphasizes the importance of children's voices in everyday school affairs, advocating for their genuine influence and impact on relevant issues (15). It also underscores democracy and participation as core values. The Estonian National Curriculum, grounded in various international human rights documents, echoes these sentiments. It aims to promote a learning environment that respects mutual perspectives, encourages free opinion exchange, and upholds human rights, equal treatment, and gender equality (16, Section 3, para. 6).

Norway and Estonia are two countries that in the past decade have generated an increasing body of literature on pupils' voices in school-based physical activity. In Estonia, studies within the Schools in Motion program offer insights into pupils' understanding of physical activity opportunities in the classroom (17), while a more recent study examined pupils' preferred activities during outdoor recess with gendered differences (18). Additional Estonian research has delved into barriers and facilitators to recess physical activity based on pupils' perspectives (19). In Norway, a few studies have focused on perceived enjoyment of pupils in physical activity interventions (20, 21), while one study explored adolescents' co-creation of health promotion measures, which made them feel resigned and dissatisfied (13). A recent study focused on pupils' experiences of physical and social affordances in Norwegian and Estonian schools (22). In the Norwegian Active and Healthy Kids program, Schmidt et al. (23) identified that classroom-based movement integration was experienced by adolescents as a different way of learning, creating feelings of both togetherness and social exclusion. However, there is limited research that specifically addresses pupils' experiences of participation in school-based physical activity. A recent report from the Active and Healthy Kids program in high school showed that pupils experienced participation in the different components of the program to a certain extent, had a desire to be heard more, and wanted their teachers to collaborate more across subjects (24).

To approach pupil involvement and participation in the school context, the theoretical perspectives were based upon Roger Hart's (25) Ladder of Children's Participation. Hart's model was used to show how pupils were able to express their voices and participate in PAL and recess according to age and context in the two countries. Hart's (25) ladder comprises eight rungs that indicate different levels of children's participation, with the first three rungs demonstrating non-participation and the remaining five showing various levels of participation. This model is useful as it can demonstrate how pupils' narratives correspond to the rungs of the ladder and can lead to discussions on how adults can act as facilitators and work with pupils at the participation level they choose according to their capacity and desire (26). Hart (25) underlines that children do not always work at the highest rungs of the ladder. What is crucial here is choice, as different children at different times choose to operate at different degrees of participation and responsibility, and adults should support the children in operating at the maximum of their abilities if they choose to do so. Criticisms of Hart's ladder include issues of power with children and adults performing in different groups and the model's disregard of contextual factors such as socioeconomic levels, which can influence children's ability to participate (27).

The aim of this study is to explore how 10- and 15-year-old pupils perceive their participation roles in school-based physical activity connected to age and the contexts of PAL and recess across schools in Norway and Estonia. To achieve this aim, the following research questions were formulated:

1. How do 10- and 15-year-old pupils experience existing structures and practices that enable them to voice their opinions on PAL and recess in selected schools in Norway and Estonia?
2. What are the 10- and 15-year-old pupils' wishes and suggestions for facilitation of their voices in PAL and recess in selected schools in Norway and Estonia?

## 2 Materials and methods

### 2.1 Study design and procedure

This article draws on data from an exploration of pupils' experiences of participation in decision-making in school-based physical activity (28). That study revealed a disparity between how pupils articulated their viewpoints on decision-making in school-based physical activity and their perception of teachers' reactions to these viewpoints. The current article concentrates on analyzing the data that focus on the facilitation of pupils' voices in PAL and recess and their perceived participation roles. The theoretical foundation for the study is related to the field of childhood studies and social constructionism where children are seen as competent social actors in research (29).

This study is based on nine focus group interviews with pupils aged 10 and 15 years (*n* = 38; 17 boys and 21 girls; 21 10-year-olds and 17 15-year-olds) conducted from January to June 2022 in two schools in Norway and three schools in Estonia (see Table 1). The same method and sample size were used in a previous article (28). Two rural and three urban schools were chosen to capture differences and similarities in how primary and secondary school pupils experienced participation roles in school-based physical activity during PAL and recess. The selection of pupils of 10 and 15 years of age was to showcase first that there is a clear decrease in physical activity from 9 to 15 years (30) and second, that older children may participate in higher rungs of participation (31). We applied the following inclusion criteria to invite schools to join the study:

• The school had focused on school-based physical activity, either through enrolment in physical activity programs or having teachers who were educated in PAL,
• The school had implemented PAL either as active lessons with academic content or as active breaks during lessons,
• The school had 10- and/or 15-year-old pupils in the school year 2021–2022.

**Table 1:** Participant characteristics.

Country	Rural/Urban	Primary/secondary education	Grade	Focus groups
Norway	Rural	1–7	5th^a^ grade	Focus group 1: 2 boys, 1 girl (10-year-olds) Focus group 2: 1 boy, 2 girls (10-year-olds) Focus group 3: 1 boy, 2 girls (10-year-olds)
Norway	Urban	8–10	9th^b^ grade	Focus group 4: 2 boys, 1 girl (15-year-olds) Focus group 5: 1 boy, 2 girls (15-year-olds)
Estonia	Rural	1–12	4th + 8th grade	Focus group 6: 3 boys, 4 girls (10-year-olds) Focus group 7: 4 boys, 2 girls (15-year-olds)
Estonia	Urban	1–9	4th grade	Focus group 8: 2 boys, 3 girls (10-year-olds)
Estonia	Urban	1–9	8th grade	Focus group 9: 1 boy, 4 girls (15-year-olds)

^a^ 5th grade in Norway is equal to 4th grade in Estonia (10-year-olds).

^b^ 9th grade in Norway is equal to 8th grade in Estonia (15-year-olds).

The invitation to participate in the study was sent to 33 Norwegian and 15 Estonian schools. Of these, five schools accepted the invitation, but further inclusion of schools was difficult, mostly due to COVID-19. The Estonian schools were part of the national program Schools in Motion (32), and the Norwegian schools were part of the regional program called Active and Healthy Kids Program (see for instance 33), or had teachers who had completed PAL education by the Centre for Physically Active Learning (34). The school principals and teachers at the schools approved the study, and written informed consent was obtained from parents/guardians and pupils electronically or on paper. In Norway, pupils were granted oral consent instead of written consent in some cases after being informed of the objectives of the study. The teachers of the participating grades were asked to choose pupils to take part in the focus groups, based on mixed genders, differing levels of physical activity and the inclusion of both outspoken and quieter pupils.

The size of the focus groups ranged from three to seven participants. The focus group discussions were audio-recorded and lasted for 45–60 min. They were conducted at the time and location most convenient for the teachers, the pupils, and the school. A semi-structured guide (see Supplementary Appendix I) was used consisting of questions such as: *What do you think the teachers could do to make you feel they listen to you more? How would you describe your ideal active lesson?* The Estonian focus groups were led by the fourth and fifth co-authors, while the Norwegian focus groups were led by the first author and a research assistant fluent in Norwegian.

### 2.2 Analysis and reflection

The data, consisting of 141 transcribed pages in Word format, were analyzed using reflexive thematic analysis (RTA) (35, 36). The following interview questions “*Can you suggest ways of doing the active lessons, active breaks,* *and schoolyard recess at school? Why? Why not?”,* *“What could you and/or your classmates do to move about more in lessons?”, and “What do you think the teachers could do to make lessons more physically active?”* were only used in this article.

Data were transcribed verbatim and translated into English by an Estonian transcription company, while the Norwegian data were transcribed verbatim in Norwegian by a trusted external person. The Norwegian transcripts were checked for accuracy against the audio files by the first author, and the English translations of the Estonian files were cross-checked for accuracy by the first, fourth and fifth authors. The analysis was facilitated using NVivo 12 software [QSR Version R1(2020)]. A central point of Braun and Clarke's thematic analysis is reflexivity (35). RTA recognizes researcher subjectivity as a means for research, highlights researcher reflexivity and argues that coding is rarely clear-cut due to the interpretation of the process (35). In this context, all authors worked in public health or education and had experience of research with children. In Norway, the first author made three visits to the primary school and one visit to the secondary school in connection with the objectives of this study. The three Estonian schools were visited once by the researchers to collect data. Providing transparency as to the authors' background and prior relations to the study schools enhances understanding of the reasoning behind the methodological choices and analysis. The data analysis followed the six steps of RTA: (a) familiarization, (b) coding, (c) generating initial themes, (d) developing and reviewing themes, (e) refining, defining, and naming themes, and (f) writing up (36). The first author became familiar with the data by reading the transcripts numerous times and making notes on paper. Each transcript was coded twice. NVivo supported coding with annotations linked to the transcripts, creating 66 initial codes. The data set was initially analyzed with the research questions in mind, focusing on pupils' experiences of existing structures and practices as well as their wishes and suggestions for facilitation of their voices in PAL and recess. The initial codes were clustered into 33 codes, which generated three main themes with eleven subthemes. The themes were reviewed against participation theory to enhance understanding of the data. The RTA as a flexible theoretical approach enabled us to inform the analysis with themes in existing research on participation theory, resulting in the development of pupils' participation roles in relation to context (PAL and recess) as well as age (10- and 15-year-olds). Braun and Clarke (36) argue that RTA as an analytic approach is on a continuum between inductive and deductive analysis. A draft was written based on the initial themes (see Supplementary Appendix II), which was read and reviewed by the second, third, and last authors, following an iterative process of going back and forth between various perspectives. This resulted in the generation of three themes with seven subthemes. All the co-authors reviewed themes, subthemes and codes to agree that they accurately reflected what the pupils expressed through written feedback. The first author wrote up the findings with input from all authors. To enhance the trustworthiness of the study, the co-authors served as “critical reviewers” throughout the stages of data collection, analysis, and manuscript writing (37). This process promoted deeper reflection and consideration of alternative data interpretations.

### 2.3 Ethical considerations

The study was approved by the Norwegian Centre for Research Data (NSD), project number 419159, and by the Research Ethics Committee of the University of Tartu, approval numbers 330/T-7 and 350/M-6. In addition to parental consent and pupils' consent or assent, all pupils were informed during the focus groups about the objectives of the study and their right to withdraw at any time with no consequences for them in relation to peers, teachers or parents. Pupils were also told that their details and interview content would be processed confidentially at grade level. The names of pupils and schools have been replaced with general descriptive categories. Therefore, there is always a risk that pupils will not recognize themselves in the researchers' findings, as we tried to examine data across several informants, and also discussed the data within the theoretical framework of Hart's ladder of participation. However, we have tried to report pupils’ accounts as accurately as possible, as the pupils' voices in this project are crucial.

## 3 Results

Three interlinked themes were formulated from the analysis, with a group of subthemes derived from each theme. The themes reveal roles of the participating child. They are presented below (Table 2) with excerpts from participants to illustrate the findings. The pupils talked about practices and structures that enabled them to voice their opinions and influence decisions on particular activities in school-based physical activity. Further, they came up with suggestions/wishes as to what could be done by both teachers and pupils to facilitate their voices in PAL and recess. The practices and structures mentioned are not always used in connection with physical activity. They could be part of another school subject but have the potential to be used in school-based physical activity.

**Table 2:** Themes and subthemes.

1. The informed and listened to a. being listened to when pupils needed a break b. being informed in advance when pupils have PAL 2. The responsible and open to teachers’ questions and facilitation a. being asked and wishing to be asked as a means of involvement b. responsibility and teacher facilitation 3. The invited to have a choice and practice participation a. activity choice b. “we had a chance to have a say” c. pupils' suggestions for moving more

### 3.1 The informed and listened to

#### 3.1.1 Being listened to when pupils needed a break

Some Norwegian pupils valued being listened to by their teachers to enable them to feel comfortable and express their need for physical activity. In 90 min lessons such as math, the pupils told their teacher that they needed a break, to take a walk around the school building, and the response was often positive, allowing the pupils to move and then come back to their work. This mostly depended on the teacher but being listened to when they needed it was considered important by the pupils to foster good relations with their teachers.

P3: For example, our math teacher is very good at giving us breaks and if we ask something like “Can we go for a walk around the building?”, she very often says “Yes” or possibly “Let's make an agreement about that then” so we can sort of move if we feel we need to. (Norwegian 15-year-olds, FG5)

The examples about being listened to regarding being active and moving in class were less prominent in the Estonian focus groups, with one group of younger pupils remembering a time when they chose to play ball games, and the teachers allowed the activities.

I: Now, could you maybe give an example of when you suggested an activity that the whole class then did?

P1: The ball fight.

I: Have you come up with something that the whole class did?

P3: “The Hunter”.

I: Yes. Anything else?

P3: The basketball, the 10-point game.

I: Do I understand correctly that these activities are all your own initiative? You suggest them and then play? Or does the teacher say that now you have to play this and that's it? The teacher says nothing?

P1: We choose ourselves.

I: (….) How has the teacher reacted?

P3: They don't react very much. (Estonian 10-year-olds, FG8)

#### 3.1.2 Being informed in advance when pupils have PAL

Pupils in one focus group at the Norwegian secondary school appreciated being informed by their teachers about what was coming up in their timetable. However, this subtheme was not mentioned by the Estonian pupils. Examples from Norway were when the teacher told the pupils when they were going to have an active break, to stretch their legs by having Just Dance or take a walk around the school building before carrying on with their schoolwork. It seemed to be important for them to have the active break or PAL written on the timetable. However, there were instances when the teachers did not inform the pupils in advance about PAL time on their schedule. This surprised some pupils when it was suddenly time for these activities.

P1: And so sometimes it's planned when they write on the timetable, so it says like leg stretching.

P2: At the start of the class, she [the teacher] often says what we're going to do in class, especially in math [….] And then she writes, for example, “sums”, “PAL” and then again “sums”.

(….)

P1: …. but sometimes it comes suddenly. There are times when they don't inform us. (Norwegian 15-year-olds, FG5)

The fact that the pupils were not prepared for what was coming up on the timetable meant insufficient time to prepare for PAL. The pupils reported that outdoor PAL lessons were often 15 min long, thus not providing enough time for the class to gather outside, move to the football field that was further down the hill from their school and have enough time to spend on the actual active lesson.

P1: You know, we often don't have that much time for it, like it's maybe kind of … 10 min.. you know, it's not often more than 10–15 min being outdoors.

(….)

P1: It's very sort of…fast: “Just do it fast”.

P2: It's a bit far to walk, isn't it? And then if you only have 10 min, then it's like…maybe four minutes until we're all together.

(Norwegian 15-year-olds, FG5)

Based on the theme analysis, the pupils appeared to appreciate instances when they were informed and listened to. However, the above examples were not the norm of how everyday school-based physical activity took place in the selected grades. The group of Estonian 10-year-olds emphasized the importance of participation through having their suggestions for activities listened to while the older Norwegian pupils highlighted the importance of taking a break when needed and being informed in advance of what would follow in their timetable.

### 3.2 The responsible and open to teachers' questions and facilitation

#### 3.2.1 Being asked and wishing to be asked as a means of involvement

Pupils in both countries were not often asked for their opinions and suggestions for activities in active lessons. However, one group of Norwegian 10-year-olds referred to an existing structure called “question box”, which involved anonymously writing about matters that affected them, e.g., puberty or friendship, on post-its and placing them in the box for the teacher later to read aloud some of the pupils' anonymous questions and try to provide answers. In Estonia, however, adolescents were generally asked about taking tests and keeping up with the curriculum. “Now everyone asks us to take lots of tests, to study a lot” (Estonian 15-year-olds, FG7). The two groups of Norwegian adolescents wished that the teachers would involve them more by asking them for their suggestions in PAL since physically active lessons could be monotonous and repetitive with standing in line, running back and forth and having only 10–15 min outdoors. Being asked and having a say in decisions could improve learning and involvement, as shown here:

P2: For example…. If the teacher had said something like “Do you have any suggestions?”, then the whole class could have come up with suggestions.

P1: Yes, because I feel we might learn more if we get to choose a little for ourselves what we want to do, because we do the same thing all the time and then there won't be that much more learning, I feel.

(…)

I: Do you want to talk to the teacher about activities, or not?

P2: We want to be involved and decide for ourselves. (Norwegian 15-year-olds, FG5)

#### 3.2.2 Responsibility and teacher facilitation

This subtheme reveals how pupils felt responsible in expressing their voices about involvement in PAL and recess and how they requested teacher facilitation since their opportunities for movement and involvement in PAL were dependent on their teachers' ability to accommodate their needs during the school day. In some Estonian schools, there was resistance from teachers to more physical activity in class due to time pressure, curriculum demands and learning goals, which eventually resulted in turning down pupils' suggestions for more physical exercise (28). However, some older Estonian pupils stated that they could ask their class teacher if they could go to the gym during recess, while younger Estonian pupils felt that the voice of the majority could have an impact on their teachers' attitudes.

I: ….And if you're thinking specifically, what could you or your classmates do to make you move about more in class?

P3: Agree on something, all of us together, and then ask together.

(….)

P1: Like during a break [recess], for example, and then the next day you ask your friends, like, let's pick an activity, and then we'll ask the teacher if everyone agrees.

(Estonian 10-year-olds, FG6)

The younger Norwegian groups suggested that they could talk to a teacher about having more physical activity in the classroom, more often active breaks or more activities outdoors or even make their requests through the formal participation structure of the pupils' council. A group of older Norwegian pupils perceived responsibility differently when they were discussing with their teachers and suggesting ways to make the lessons more physically active. The pupils talked about how they preferred to have their teachers ask first and be open to their suggestions instead of them suggesting activities at a time when the teachers might have been busy or generally not available to consider the suggestions.

P3: I also kind of feel it's a bit easier if the teachers ask us “Do you have any suggestions?” than if we make suggestions to them, because if we do, then we have to sort of find a time that fits… suggest [activities] suddenly when it doesn't fit in or there isn't time [in the lesson]…. I feel like it's better if they ask us, because then you feel like they care a bit. If we suggest it first, they might find it a bit annoying, you know.

P1: You might feel you're going on at them a bit. So, it would be better to have some [free] time in class to have a break and they could listen to us too.

P3: We don't want them to be annoyed or anything like that…, they can decide when it's appropriate to get these suggestions [from us]. (Norwegian 15-year-olds, FG5)

For the older pupils, one powerful element of participation was teacher facilitation, meaning that teachers were receptive to pupils' suggestions, and did not try to control them but provided space, motivation and guidance when needed, acting as equal partners. This subtheme mostly reappeared in the Estonian focus groups of both ages. One group of Estonian adolescents discussed how they would like their teachers to be more open to their requests for having more physical activity in the classroom instead of traditional teaching and to have them organize activities where both teachers and pupils motivate each other, and the teachers act as active role models for their pupils:

P3: She could try before she says there's nothing you can do with our class, but try and then say … And if it doesn't work out or nobody learns anything, then let's not do it anymore. But she could still try.

(…)

I: If you're thinking about active breaks, what would you recommend to the teachers?

P3: For them to get involved themselves and to invite students to join in. If they themselves say “go, go”, and at the same time they're sitting somewhere in the classroom, then you don't really want to get moving either.

P2: To figure out what to do themselves. Not like when you go and suggest something to do, they reply: “Well, do it yourself”. They should talk to the teachers about it, so the students have an idea..

P1: They could organize it themselves. They're always saying: “Do it yourself”, but they don't take part in anything. (Estonian 15-year-olds, FG9)

Overall, the above results show that although asking for pupils' opinions about PAL and recess was not established as an everyday practice, the existing democratic practice of the “question box” along with the desire of the younger and older Norwegian pupils to be asked shows the importance of creating spaces at school that facilitate participation. With regards to responsibility, we found that younger pupils in both countries were aware of their responsibility to express their requests for more exercise and breaks and their suggestions focused on asking their teachers about it either individually or as a group, as exemplified by the Estonian younger group. For the Norwegian adolescents, responsibility included having a space to ask questions that was offered first by the teachers. Pupils were also open to teacher facilitation, especially Estonian younger and older pupils. Teachers were seen as key enablers of participation in the school setting (38).

### 3.3 The invited to have a choice and practice participation

#### 3.3.1 Activity choice

Pupils aged 10 and 15 talked about what practices and structures in school enabled them to participate in decisions on PAL. In this subtheme, pupils of both age groups mentioned having a choice in which activities to perform when drawing an activity from an activity box, raising their hands to agree to a proposed activity, having a “task wheel” in class with activities they could choose, or using popsicle sticks to draw the name of a pupil to decide on the active break during PAL as in the examples below: these practices were informal outlets for pupils to express their opinions and invitations to practice participation.

P3: When we have [active] breaks, the teacher goes on YouTube and draws a popsicle stick from a coffee cup with the name of a pupil [written on it], who gets to pick which Just Dance or Brain Break activity we're going to do. (Norwegian 10-year-olds, FG2)

P1: We had this box in our last school that had different activities inside it. We pulled one out of there and we did it. (Estonian 15-year-olds, FG7)

Additionally, the practice of using activity leaders was implemented in three out of the five schools of the study. Activity leaders are one of many physical activity initiatives to invite pupils to be peer mentors in activities for their classmates. The organization can vary from school to school, but generally, it stems from pupils' interests in activities, and the mentors organize and supervise the activities for their peers, usually during recess. As a group of younger pupils discussed, the use of activity leaders was a way to practice participation and decide on the activities in which they would guide their classmates in recess, thus consisting of a more formal participation structure.

P2: Among the activities, there is dodgeball, chess, dancing, longball, Jenga, mini football. And sandboxes. Basketball and frisbee.

(….)

P2: To be an activity leader, you should go out for recess five minutes before the others and put on a vest like this. You also take the activity [equipment] you have chosen and place it where you want it since you were told where to have it. You wait for the others to come and you prepare the game for them… You can also join the game except for dancing. Then you're not allowed to join even if you're an activity leader.

I. And how do you decide on these games?

P2: We decide which games to play by writing on a sheet of paper…. If we feel like dodgeball, we write it there. We also write our names next to the activities we want to do … you have to register on which day you want to join.

(Norwegian 10-year-olds, FG1)

#### 3.3.2 “We had a chance to have a say”: the pupils' council as a structure of participation in school-based physical activity

Pupils in both countries talked about the existence and the functionality of pupils' councils at their schools. Pupils' councils are not directly related to school-based physical activity but can handle requests or matters related to physical activity. Furthermore, the pupils' councils in this study do not operate as part of the school-based physical activity programs but are part of the school culture. They are typically comprised of pupils elected by their peers to represent their needs and interests at their school (27). Pupils' councils are viewed as arenas for pupils' meaningful participation in decision-making, since they are often pupils' first point of contact with democratic processes for promoting their student rights (27).

Based on the participants' accounts, there were times that the pupils' councils worked efficiently on physical activity cases pertaining to decisions about new games or buying new equipment, as shown in this quote:

P3: We have something called the pupils' council. There are post-it notes where we can write our suggestions about what we should have at school. Then, those who are in the pupils' council take our requests and discuss them in a meeting.

(….)

All the pupils can…when it gets closer to the meeting or at any time…write a game, or something they would like more of, or activities for the pupils or anything like that and they hang their requests on a white poster that we (members of the pupils' council) discuss in front of the whole class, the day before the meeting. And it goes like this, for example this toy, if it costs a lot of money, we put it on the red list, if the price is ok, then on the green. If it's cheap and useful, then it's green. And if it's in between, we put it on yellow. (Norwegian 10-year-olds, FG2)

(….)

P2. It may happen that someone has a suggestion written on a post-it like for example “I want to have “The Champion of Champions” in our school or the “Celebrity Task Force” ^1^ …And then this is an activity suggestion and we consider whether they should do it or not. So, it could be a no, perhaps, or we will look at it. (Norwegian 10-year-olds, FG1).

At other times, the pupils' council existed as a decision-making organ at school but was not effective according to the pupils, who expressed their dislike of the way it operated.

P1: We had this group that discussed other things, about a development plan and so on, but *we had a chance to have a say—*about 10 students or so—they got a say in what we wanted for the next school building. It was decided.

P2: Schools could introduce more plans about what they intend to do. So that we could understand what they're doing. Then you could also think about what else could be done.

P2: They leave everything to the last minute. And then they finally say no.

I: Don't you have anyone from the pupils' council who can have a say? The school board or something?

P1: (…) The pupils' council kind of operates in the background.

P2: They do other things. They don't deal with activities.

P1: They organize events. We don't have a school that is keen to have theme days or things like that.

(Estonian 15-year-olds, FG7)

#### 3.3.3 Pupils' suggestions for moving more

The pupils' responses revealed that they wanted more movement in the classroom. They suggested spending more time outside and performing more enjoyable PAL tasks outdoors, having more frequent active breaks, having more frequent and varied PAL lessons, going on field trips during lessons, and playing more games combined with academic content.

P2: Or something like a children's game, only that we involve the academic side, then.

(….)

P3: It could, for example, be a bit like that … we had something like that … I don't know, some kind of game where you can kind of lose, and the ones who lose have to go out and, for example, get a question from the teacher, then they must answer correctly to join the game again. (Norwegian 15-year-olds, FG5)

With regards to recess, pupils wanted their schools to buy more equipment like rackets or balls, have more natural areas and open facilities for adolescents like gyms or sports centres to go to during recess time and more often have dance breaks during recess. One 4th grade pupil said:

P1: There could be more of those dance lessons.

I: Uh-huh. How much more would you like there to be?

P1: I'd say Wednesdays and Thursdays, for instance. (Estonian 10-year-olds, FG6)

Based on the data analysis above, it was important for pupils to have a choice in the activities and practice participation in decisions on PAL and recess. The results show both informal and formal structures and practices of participation. Both age groups in both countries talked about the importance of these avenues to participation even though at times they expressed frustration at the lack of functioning of the pupils' council at their school. Regarding the pupils' suggestions for moving about in PAL and recess, both age groups in both countries suggested examples of more physical activity in class and recess, buying more equipment for recess, and having more open facilities for adolescents. Expressing their views and wishes about what they would like to have in PAL and recess was an opportunity to practice everyday participation.

## 4 Discussion

This article sets out to explore how the 10-and 15-year-old pupils perceive their participation roles in school-based physical activity connected to age and the contexts of PAL and recess in Norway and Estonia. Our RTA developed three main themes based on pupils' experiences of existing school structures/practices and suggestions for participation in PAL and recess. These themes revealed aspects of the pupils' role as a participant in school-based physical activity and will now be discussed in light of Hart's (25) ladder of participation, first in the context of PAL and recess and second in relation to age.

### 4.1 Pupils' perceived roles in participation in the contexts of PAL and recess

In this article, roles in participation were presented through existing participation structures/practices and pupils' suggestions for facilitation of their voices in PAL and recess. Roles in participation vary with context and age. PAL and recess contexts differ in structure and participation. PAL in the form of active lessons and active breaks can take place both in the classroom and outdoors. PAL is teacher-led, meaning that the teacher initiates the activities taking place in the classroom. In this context, participation of pupils' voices is often restricted by the limited time of PAL sessions “and then if you only have 10 min” and the repetitive nature of PAL. In active breaks, e.g., walking around the school building or drawing popsicle sticks for Just Dance breaks, the findings show that pupils experienced freedom of choice in activities. However, permission and organization of the activities were granted by the class teacher, not by the pupils, leading to the question of whether this is genuine participation. According to Hart's (25) ladder of participation, the example of active breaks could resonate with rung 4, *assigned but informed*, where the active breaks are directed by the teachers but the pupils are fully informed about why they have the specific active break (interrupts sitting, makes them feel energized before continuing with the lesson or fits with the learning topic of the day) and have ownership in the sense that they can choose what exercise to do, e.g., “jumping jacks” or “high knees”. In a health education context, Simovska (39) concluded that shared responsibility and collaborative effort led to an enhanced sense of ownership among students regarding their learning. Furthermore, the above practices along with the question box, raising hands, and drawing from an activity box based on choices of activity constitute informal spaces of participation that according to research tend to be valued more by children than formal spaces as they represent everyday experiences of citizenship (38).

Also, in the context of PAL, a group of older Estonian pupils wished for teacher facilitation in organizing activities in PAL, more movement in the classroom in contrast to the existing sedentary lessons and teachers engaging in the activities and acting as role models for their students. Quarmby et al. (8) reported similar results with teachers facilitating learning and social interaction between peers by introducing PAL in their classes. Incorporating physical activity in class compared to traditional sedentary lessons can create a shift in pedagogy, where the teacher's role is oriented towards pupils' prerequisites and interests as the starting point for teaching (8). As seen in previous research, this may position PAL as a constructive approach to pedagogy, where pupils are activated in their learning process (40), which can redirect the teacher's focus from ensuring that pupils follow rules and managing their behaviour (8, 41). Here, it is worth mentioning that teachers might be restricted by other factors such as lack of creativity, and non-availability of resources (42, 43), which may have a domino effect on the extent to which pupils can become involved in PAL. A recent study found that many teachers lacked confidence and competence in PAL activities, potentially leading to frustration, resistance, and an absence of a consistent approach to integrating movement (39).

Recess, as the second context in our study, is considered autonomous and child-led, with pupils having a break from lessons and the freedom to decide how they want to spend their free time. Yet, rules around safety, weather and seasons, availability of equipment and schoolyard affordances may influence the way of doing recess (22). One of the ways that pupils demonstrated participation in recess in three schools of the study was through the use of activity leaders. In an evaluation of a study of participation in school-based physical activity, teachers and students emphasized the need to find ways to empower students (44). The results indicated low participation of girls and adolescents. Among the proposed recommendations was a suggestion to form a group of student leaders to better address student needs and thus have more equal collaboration between teachers and students as a shift from the traditional hierarchy often encountered in schools (44). Although student leaders can minimize power differences between pupils and teachers, it is worth noting that using activity leaders is predominantly an intervention organized by the school, where pupils have predefined margins within which they can take decisions, motivate their peers and choose activities. Further, the findings of the current study showed that the pupils' councils were another arena of participation where pupils could raise matters of equipment and decide on activities and games during recess. With regards to Hart's ladder of participation, pupils' councils and activity leaders are both placed higher on the ladder, belonging to rung 7, *child-initiated and directed*, where pupils' play initiated by activity leaders and decisions reached by the pupils' council are child-led, with adults acting as observers and facilitators. With recess being child-led compared to the teacher-led PAL and pupils' councils and activity leaders having predefined rules, there is a risk that any pupil involvement could be considered *tokenistic* (rung 3), meaning that teachers or any adults in charge may comply with rules about listening to pupils' views but eventually ignore them (45). The pitfall with pupils' councils is that they are miniatures of existing institutional practices (46), thus constituting formal spaces of participation. Participation in these spaces is therefore more suitable for pupils who understand the rules and have a skillset to contribute to the processes involved (47). In other words, these spaces will benefit and utilize the skillsets of pupils who are able to participate regardless, and of those who behave like mini-adults (46, 48). While formal participation structures might reduce free play/free choice during recess by being imposed by adults although mediated through the activity leaders or peers, it is important not to view formal participation structures as “bad” and informal ones as “good”, since the former are recognized as important in facilitating participation by children whose schools did not have pupils' councils (38, 49).

In general, PAL and recess do not have an equal voice in participation, with recess ranking higher in Hart's ladder of participation than PAL by default. Does this mean that PAL should be child-led and autonomous like recess? Pupil participation should not be misconstrued as pupil determination, a common misconception that falsely equates participation with pupils having the final say (50). Even when pupils engage at higher levels on the participation ladder, bringing changes to school-based physical activity, they still require the collaboration of respectful and equal partners. Here, teachers serve this role, providing support, presenting challenges, and creating a safe environment where pupils can experiment with their ideas (51).

Overall, the findings revealed that even though PAL with its active lessons and active breaks was predominantly teacher-led, pupils felt ownership of the activities by being able to choose them and they requested more engaging and facilitating teachers who would act as role models for them. Recess is viewed as autonomous play and free time from lessons, which basically implies higher rungs of participation. In this study, participation was realized in an informal manner through freedom of choice in PAL activities, but also through the formal practices of activity leaders and pupils' councils, which needed to be handled carefully by adult facilitators to avoid tokenistic participation.

### 4.2 Pupils' roles in participation in school-based physical activity by age

The results reveal more detailed descriptions of participation from 15-year-olds than 10-year-olds. This could be attributed to the view of older children as competent participants making participation challenging for younger children who express their views to a lesser extent about the matters that concern them (52).

Furthermore, the findings of this study suggest that pupils' perceived participation roles in school-based physical activity can vary by age. The results showed that the 10-year-olds appreciated having a choice of activities in PAL, and they had numerous ideas on how to be more active during school hours. For instance, existing participation structures such as the pupils' council could be used to influence decisions about buying new equipment and introducing new games during recess. The younger Estonian pupils grouped together to tell their teachers that they wanted more physical activity and made suggestions about it. The social support that they found in their classmates empowered them to request more physical activity from teachers, in whom they occasionally met resistance. The role of social support in empowerment is in line with a previous study highlighting that group activities leading to personal empowerment were a significant factor in the success of a physical activity intervention (53). Pupils' awareness of their responsibility in class started in the younger grades and continued in the older grades.

For the 15-year-olds of the study, elevating their voices and feeling involved in PAL and recess meant being listened to when they needed a break from sitting, being informed in advance about PAL, being asked to offer their suggestions for activities and being able to practice participation during PAL and recess. Some older Norwegian pupils felt that their teacher listened to them by addressing their need for movement after a long sedentary lesson. For Lundy (45), “voice is not enough”; it should be guaranteed that children are provided with opportunities to express their views to individuals, such as teachers in our case, or bodies with decision-making responsibilities. Additionally, a group of Norwegian adolescents found it important to be informed about upcoming active breaks during the school day and to have the PAL time written on their timetable. Granting children and young people access to information is deemed crucial in enabling them to express an educated perspective (54). Although it is not explicitly mentioned in Article 12 of the UNCRC, the 2009 General Comment of the UNCRC underscores the importance of mutual information exchange and dialogue in the participation process (45).

In both countries, pupils wanted teachers to ask them for their opinions and suggestions for physical activity, but teachers rarely asked them (28). This can be understood as a question of space, i.e., opportunities for involvement and suitable conditions for children to feel safe to express their views (45). The first step is to invite and encourage children's views on a relevant topic rather than receiving their input at random (45). The pupils in the present study felt that asking was an essential means to elevate their voices, with some younger pupils highlighting the responsibility of having to make suggestions that would make them more physically active in class and recess. Older pupils perceived responsibility differently, such as finding the right time and space to offer their activity suggestions to teachers. Responsibility has always been a crucial aspect of children's participation in research, particularly in relation to power sharing (55). Shier's model highlights that at its highest level, decision-making involves both children and adults, unlike Hart's model, which implies that children can make decisions independently. In Shier's model, children are actively involved but may not have final decision-making power. This emphasizes the commitment of adults to share some of their power (55). In this study, the pupils' requests for teacher facilitation or power sharing reflect this concept. Shared power can lead to shared responsibility, at least in some aspects of decision-making (55).

In the theme *The invited to have a choice and practice participation,* we found that pupils of both ages and countries were aware of practices and structures that enabled them to have a choice in physical activities and a say in decision-making. Both older and younger pupils provided examples of PAL activities that underscored the importance of having choice as a key aspect of their role in participation in school-based physical activity. A review by Martin (56) emphasized that pupils greatly valued their autonomy and choice when participating in physical activity. When pupils were granted the autonomy to opt for activities that appealed to them, they were more inclined to participate as these activities were perceived as inclusive and respectful of their needs, abilities, skills, and activity preferences (56). For some Estonian adolescents, the pupils' council could be an organ for decision-making, but it was also ineffective at times. In order for participation to be effective, there must be opportunities to exercise it, enabling both teachers and pupils to gain practical understanding of what participation entails (26).

Ultimately, with regards to roles of participation by age, the themes mainly reflected the experiences of adolescents. The 10-year-olds expressed appreciation over the functionality of the pupils' council, having a choice in PAL activities, and offered many ideas for increased physical activity during school hours. The 15-year-olds sought to be heard when needing a break from sitting, be informed about PAL in advance, be consulted for activity suggestions, and have the opportunity to participate in decision-making during PAL and recess. Their roles involved a higher degree of responsibility and frequent requests for teacher facilitation compared to the 10-year-olds.

### 4.3 Strengths and limitations

By drawing on RTA of pupils' experiences in school-based physical activity, the results of this study are context-specific to the five selected schools. The transferability of the results may thus be limited to similar contexts, and one cannot assume the generation of similar results in the schools enrolled in Schools in Motion, Active and Healthy Kids Program and Centre for Physically Active Learning. However, the inductive approach to the data and the robust stages of RTA that necessitated a thorough review of codes and themes by the co-authors ensured a rich and varied representation of pupils' experiences of their roles in participation in two countries and two age groups. An additional constraint was that the study took place immediately after the COVID-19 restrictions had been eased. This had direct implications for the number of schools recruited, the frequency of school visits and the use of diverse research methods. The study also has a number of strengths. As far as the authors are aware, this study is the first to utilize examples from both 10-year-old and 15-year-old pupils in schools in Norway and Estonia to identify different roles in participation by age and context in school-based physical activity. An additional strength is the two-country approach to exploring pupils' perceived roles in participation, which can identify common trends and crucial disparities in different countries (57).

## 5 Recommendations and concluding remarks

This empirical study referred to the few instances in which pupils were heard or where their views were given priority. There is limited research involving pupils' voices on school-based physical activity, especially PAL, with few studies exploring pupils' experiences and perspectives (8). This article explored how 10- and 15-year-old pupils perceived their participation roles in school-based physical activity connected to age and the contexts of PAL and recess in various schools in Norway and Estonia.

Overall, the results highlight that pupils participated through informal and formal practices and structures of participation in teacher-led PAL and active recesses. Based on the more formal teacher-led PAL practices, this study recommends that teachers structure teaching with democratic spaces for pupils to exercise their freedom of expression and rights to participate in school-based physical activity. To do so, our study first recommends that pupils' participation in PAL is dependent on context and age. This means that PAL, as a democratic space, should consider that while younger pupils may need clearer rules to participate, older pupils may need to be more autonomous in their participation. Although Gådin et al. (58) found that pupils as young as seven can be involved in change proposals and goal setting and actively participate in creating change in school-based physical activity, our findings propose that it is important that teachers remain in charge of decisions. In a democratic sense, this means that it is the teachers who should decide the activities and the educational aims; however, pupils can, to various degrees, contribute their opinions. In our findings, the 10-year-olds presented numerous ideas on how to be physically active at school, valued the functionality of the pupils' council and took responsibility for requesting more physical activity.

Additionally, our findings indicate that older pupils described more examples of participation than younger pupils in school-based physical activity. Not only did they describe more examples, but they also showed a higher degree of responsibility and regular requests for teacher facilitation. This difference could be attributed to adult notions of older pupils being more competent and mature enough to participate. Indeed, this brings us to this study's second recommendation, which is that pupils' participation in PAL and recess should start with their interests and prerequisites and prioritise a sense of ownership. This is attributed to an asymmetrical relationship between adults and children in the sense that it is the teacher who is responsible for pupils' participation. For older pupils, it might be to decide on an activity or to contribute to how the activities can be designed; for younger pupils, it might be when they need to be physically active. A choice of activities, offering ownership of the activities, and teacher facilitation of pupil participation in school-based physical activity are key in the contexts of PAL and recess.

Further, this brings us to our third recommendation, which is that teachers should structure PAL to provide a safe and sound environment for pupils to share their opinions. An important distinction is that pupils' opinions should not always be granted, but they should always be taken seriously. Taking pupils' opinions seriously means that the physical activity should include a variety of intensities and activities so that all pupils feel a sense of ownership in being active. This also means teachers should balance the relationship between support and challenges in different activities. As such, activities that offer pupils a space of autonomy might be helpful for them to participate. For instance, this may mean activities where pupils can decide the intensity or duration themselves. Indeed, PAL allows pupils to practice democratic processes because they must make active value choices, collaborate with other pupils and relate to the teacher's activities. Our fourth and final recommendation is that PAL activities should be structured with opportunities for pupils to practice rules and specific skills. This may, for instance, mean that teachers should not only be concerned with pupils' academic learning but also with their participation in the activities and how they collaborate and treat each other.

If done properly, our study indicates that PAL offers a space for pupils to participate in and contribute their voices to teaching and can thus offer a space for pupils to learn about democracy. Using PAL to learn about contribution and participation may make sense as learning about democracy and gaining the confidence and ability to do so takes practice; it cannot be taught in theory (25). As such, we conclude that pupils, being relatively marginalized in society, require adult support to cultivate and maintain participation. However, it should be noted that their degree of participation is different in PAL compared to recesses, where they have a larger degree of participation. Moreover, it is not a given that teachers inherently know the appropriate level of support or can naturally establish co-productive practices with youth (59). Therefore, there is a future need to guide and train teachers to effectively assist pupils in developing the skills necessary for active participation in school-based physical activity.

## Data Availability

The datasets presented in this article are not readily available because they contain data from underaged, where strict ethical caution regarding GDPR apply. Requests to access the datasets should be directed to corresponding author. Requests to access the datasets should be directed to Eirini Pardali, eirini.pardali@usn.no

## Footnotes

^1^The champion of champions *(Mesternes mester)* and Celebrity Task Force *(Kompani Lauritzen)* are both Norwegian TV shows with exercises and cooperation tasks.
