# Supplementary material for: Pupils' participation roles in school-based physical activity in the context of physically active learning and recess: experiences from Norwegian and Estonian primary and secondary school pupils
Source: Front Sports Act Living. 2025 Feb 24;7:1514764. doi: 10.3389/fspor.2025.1514764 (PMC11891175; doi:10.3389/fspor.2025.1514764)
Supplement: Supplementary file 1 [file Table1.docx]

**Supplementary Appendix I**

TABLE Interview guide

| Introduction, about two minutes | Informal greeting and brief description of the aim of the talk: introduction of the topic, clarification of the purpose and ethical aspects  Explain the rules of the interview:   - One person talks at a time. - Everyone can have their own opinion and it is okay if opinions do not agree.   Everything that is discussed during the interview will only be known to the pupils/people present in the interview. |
| --- | --- |
| Topic 1 | Can the pupils share their views and suggest ways of conducting active lessons, active breaks and recess? |
| Questions, about 10 minutes | We will now talk about ways you share your views about how active lessons, active breaks and school recess should look like and you can make suggestions so that the activities agree with your expectations and you are more listened to.  Do you say in class what you like or not during the active lessons, active breaks and recess? Why/Why not? How?  Were you able to participate and decide on what you wanted to do in active lessons, active breaks and recess?  What has prevented you from sharing your views at school?  Can you suggest ways of doing the active lessons, active breaks and recess in the classroom/at school? Why? Why not?  Do you want to be more involved in how to move more in lessons? Why/Why not?  What could you and/or your classmates do to move about more in lessons? |
| Topic 2 | How does the school listen to pupils and what should the school do to take their opinions more seriously? |
| Questions about 10 minutes | Now you can share your thoughts on how the school listens to your views and what the school could do to take your opinions more seriously.  Can you give me an example of a time you suggested an activity that you did together in the class?  How did the teachers respond?  What did they do?  Do the teachers listen to you? Why/why not?  Do you feel that the teachers listen to your preferences when choosing physical activities? Why/Why not?  What do you think the teachers could do to make lessons more physically active?  What do you think the teachers could do to make you feel they listen to you more? |
| Closing questions, about 10 minutes | Now you can share with me what your ideal active lesson, break and recess could be like.  Imagine being in charge of the whole school for one day…  How would you describe your ideal active lesson?  How would you describe your ideal active break? How would you describe your ideal recess? |
| Summary and end of the interview, about 5 minutes | Would you like to tell me anything else?  Thank you for participating in the interview. |
