# Supplementary material for: Pupils' participation roles in school-based physical activity in the context of physically active learning and recess: experiences from Norwegian and Estonian primary and secondary school pupils
Source: Front Sports Act Living. 2025 Feb 24;7:1514764. doi: 10.3389/fspor.2025.1514764 (PMC11891175; doi:10.3389/fspor.2025.1514764)
Supplement: Supplementary file 2 [file Table2.docx]

**Supplementary Appendix II**

TABLE Themes, code labels and selected data extracts

| RQs | Themes | Subthemes | Codes | Example data extracts |
| --- | --- | --- | --- | --- |
| RQ1: How do 10- and 15-year-old pupils experience existing structures and practices that enable them to voice their opinions on PAL and recess in selected schools in Norway and Estonia? | The informed and listened to | Being informed  **Being listened to^[[1]](#footnote-1)^**  Written on the timetable |  | P1: In the Norwegian lesson, you know, when we have that teacher and then, for example, another pupil asks “Can we have leg stretches, then?” and then the teacher says “Yes, do you want to stretch your legs?”, the whole class just shouts “Yes”, because everyone wants it.  **Norwegian 15-year-olds, FG5** |
|  | The responsible and open to teachers’ questions and facilitation | Being asked as part of existing structures and practices | Being asked by the teachers  **Digital media as a means of participation**  Question box | P1: We have ... Teams, so we can send messages to the teachers. We haven’t sent a message about the physical activities, but we’ve sent a message about other things if it’s like there’s homework we’re kind of wondering about, or... uh, if we’re like ... going to hand something in, and then we’re not sure when the deadline is, or if we can’t find an assignment, or something like that, then we can send them a message (on Teams), or by email….  P2: We have the possibility to contact them, at least.  **Norwegian 15-year-olds, FG5** |
|  | The invited to make a choice and practice participation | Activity choice | Activity leaders  **Box of activities to draw from**  Drawing names of pupils to decide about active breaks  Inclusive activities  Post-its with questions  Raising hands  Task wheel | I: Could you suggest to a teacher how to organize a class with a nice long break? Or an activity to cheer you up?  P1: We had this box in our last school that had different activities inside it. We pulled one out of there and we did it.  **Estonian 15-year-olds, FG7** |
|  |  | Pupils’ council as structure of participation in school-based physical activity |  | I. You mentioned that there is a student council? Can you explain a little about that?  P1. I’m on it. I can do that. Ehh. Then it’s like the others in the class write a note and say what they want for the school. E.g. a new football for this and that class. Or they want more markers throughout the school in all classrooms.  **Norwegian 10-year-olds, FG1** |
| RQ2: What are the 10- and 15-year-old pupils’ wishes and suggestions for facilitation of their voices in PAL and recess in selected schools in Norway and Estonia? | The informed and listened to | Wishing to have it on the weekly timetable |  | P1: Or in a way, that the teachers could give us a plan for when PAL should be. On the weekly schedule, it can say, in a way*, English, PAL* just under there, so we know we have PAL that day.  **Norwegian 15-year-olds, FG5** |
|  | The responsible and open to teachers’ questions and facilitation | Asking the teachers as pupils’ responsibility | **Pupils suggest active breaks to teachers because they might not have thought of that**  The voice of the majority | P1: I think it’s actually because we asked about it, because..., like... she (the teacher) didn’t usually do it, but when we asked about it, then she kind of let us do it, you see (have active breaks during lessons).  P2: Yeah, because she’s like, well, that’s the way she is… I think maybe the reason why she doesn’t even, maybe because she just hasn’t thought about it, like.  **Norwegian 15-year-olds, FG5** |
|  |  | Wishing to be asked by the teachers |  | P1: There was someone who had made some suggestions, and then... yes... the teacher is open to accepting suggestions.  I: Does the teacher seem open to suggestions?  P2: We don’t usually do that, no. But if the teacher had done it, we would certainly have made suggestions.  **Norwegian 15-year-olds, FG4** |
|  |  | Teacher facilitation | Engaging teachers  **What the teachers could do** | I: I understand there was an ice track on the top of the hill, which was hard and dangerous. Okay. What do you think the teachers, in general, could do to make active classes more physically active? What could they do?  P3: Make a new game with the students who are sitting too much.  **Estonian 10-year-olds, FG8** |
|  | The invited to make a choice and practice participation | Pupils’ suggestions for moving | Active break for the whole school  **Be inclusive**  Box open for activity suggestions  Dance Tuesday as it used to be  Learn from a variety of activities that the pupils can add to  Other ways of being active  Quiz combined with physical activity  Sports events  Think of games and try them out | P3: We used to have an extra thing, where everyone had to go around the building, before they could go inside.  I: It’s like an entrance ticket.  P3: To move a bit.  I: But why do you think that was done?  P3: So that the ones who were sitting in one place, so that they’d move.  I: To get everyone moving a bit. I see.  P2: The ones who played about.  I: Do they have to do a lap around the building too? Is that what you meant?  P1: Sometimes, if there’s no one in line, you have to look around the building to see if they’re there.  I: So you have to go looking for the others? I see. Ah, you line up before you come inside?  P1: Uh-huh.  **Estonian 10-year-olds, FG8** |

1. The example data extract provided comes from the highlighted subtheme or code. [↑](#footnote-ref-1)
